# Diverse growth rates in Triassic archosaurs—insights from a small terrestrial Middle Triassic pseudosuchian

**DOI:** 10.1007/s00114-024-01918-4

**Published:** 2024-07-11

**Authors:** Nicole Klein

**Affiliations:** Institute of Geosciences, Paleontology, University of Bonn, Bonn, Germany

**Keywords:** Poposauroidea, Late Anisian, Parallel-fibered tissue, Growth marks, Low growth rate

## Abstract

The small pseudosuchian *Benggwigwishingasuchus eremacarminis* was found in Anisian (Middle Triassic) marine sediments. Neither the skeleton nor osteohistology or microanatomy shows any secondary aquatic adaptations, and a dominantly terrestrial lifestyle of this new taxon is evident. Bone tissue consists of a scaffold of parallel-fibered matrix, which is moderately vascularized by small, mainly longitudinal primary osteons. The innermost cortex is less densely vascularized and more highly organized. No parts of the cortex contain any woven bone. The cortex is regularly stratified by annual growth marks. Bone tissue and growth pattern indicate an adult individual that has had slow growth rates throughout its ontogeny. Tissue type, slow growth rate, and inferred low resting metabolic rate of *Benggwigwishingasuchus* are similar to that of crocodylomorphs but differ from that of *Sillosuchus* and *Effigia*, poposaurids to which *Benggwigwishingasuchus* is related based on phylogenetic analyses. However, according to current knowledge, growth rates in early archosaurs are more likely influenced by body size and environment than by phylogeny. *Benggwigwishingasuchus* is thus another example of unpredictable variability in growth rates within Triassic archosaurs.

**Supplementary Information:**

The online version contains supplementary material available at 10.1007/s00114-024-01918-4.

## Introduction

The recently described *Benggwigwishingasuchus eremacarminis* was found in pelagic deposits laid down in the late Anisian (Middle Triassic) of the eastern Panthalassan Ocean (Smith et al. 2024). The sediments belong to the Fossil Hill Member of the Favret Formation (Favret Canyon, Augusta Mountains, NV, USA). The locality is dominated by abundant ammonoids and ichthyosaurs (Sander et al. 2021). The largely complete and partially still articulated postcranial skeleton of *Benggwigwishingasuchus* is preserved in a strong opisthotonic posture, which together with its completeness suggests minimal post-mortem transport. Morphology and femoral bone compactness shows no skeletal adaptations associated with a secondarily aquatic lifestyle (Smith et al. 2024).

Bone histology can reveal changes in bone development and bone structure, indicating a change toward a secondary lifestyle before morphological changes might have manifested (Ricqlès and Buffrénil 2001). Archosaur osteohistology is further important in an evolutionary context. High growth rates seem to represent the plesiomorphic condition in archosaurs (Botha-Brink and Smith 2011; Legendre et al. 2016; Ricqlès et al. 2021) but changed to a spectra of slow and fast growth rates, indicating manifold growth strategies in various pseudosuchian taxa. However, this diversity seems not to be related to further phylogeny (Buffrénil et al. 2021a; Ricqlès et al. 2021; Botha et al. 2023) although some trends can be observed. Avemetatarsalia generally show high growth rates throughout ontogeny, whereas Pseudosuchia show a mixture of fast (e.g., *Batrachotomus*, *Fasolasuchus*, ornithosuchids), medium (e.g., phytosaurs and aetosaurs), or slow growth rates (e.g., crocodylomorphs) (see Suppl. Table for references). Fast growth rates are more often observed in large-bodied taxa, whereas slow growth rates often occur in small bodied taxa (e.g., Botha-Brink and Smith 2011; Klein et al. 2017; Botha et al. 2023). Taxa with fast growth rates show woven bone and high vascular density resulting in a woven-parallel complex throughout most of their ontogeny. Taxa with a medium growth rate grew with a woven-parallel complex in early ontogeny and switched then to a parallel-fibered matrix and thus to a slower growth rate. Slow-growing taxa grew solely with parallel-fibered and/or lamellar-fibered matrix throughout their ontogeny. This high diversity of tissue types is restricted to Triassic taxa; from the Jurassic onward only slow growing crocodylomorphs and fast growing avemetatarsals exist.

This study describes the humeral bone tissue and microanatomy of *Benggwigwishingasuchus eremacarminis* to possibly detect a secondary aquatic adaptation and to discuss the growth rate of this Middle Triassic pseudosuchian in the light of evolutionary trends within archosaurs.

## Material and methods

**Fig. 1:**
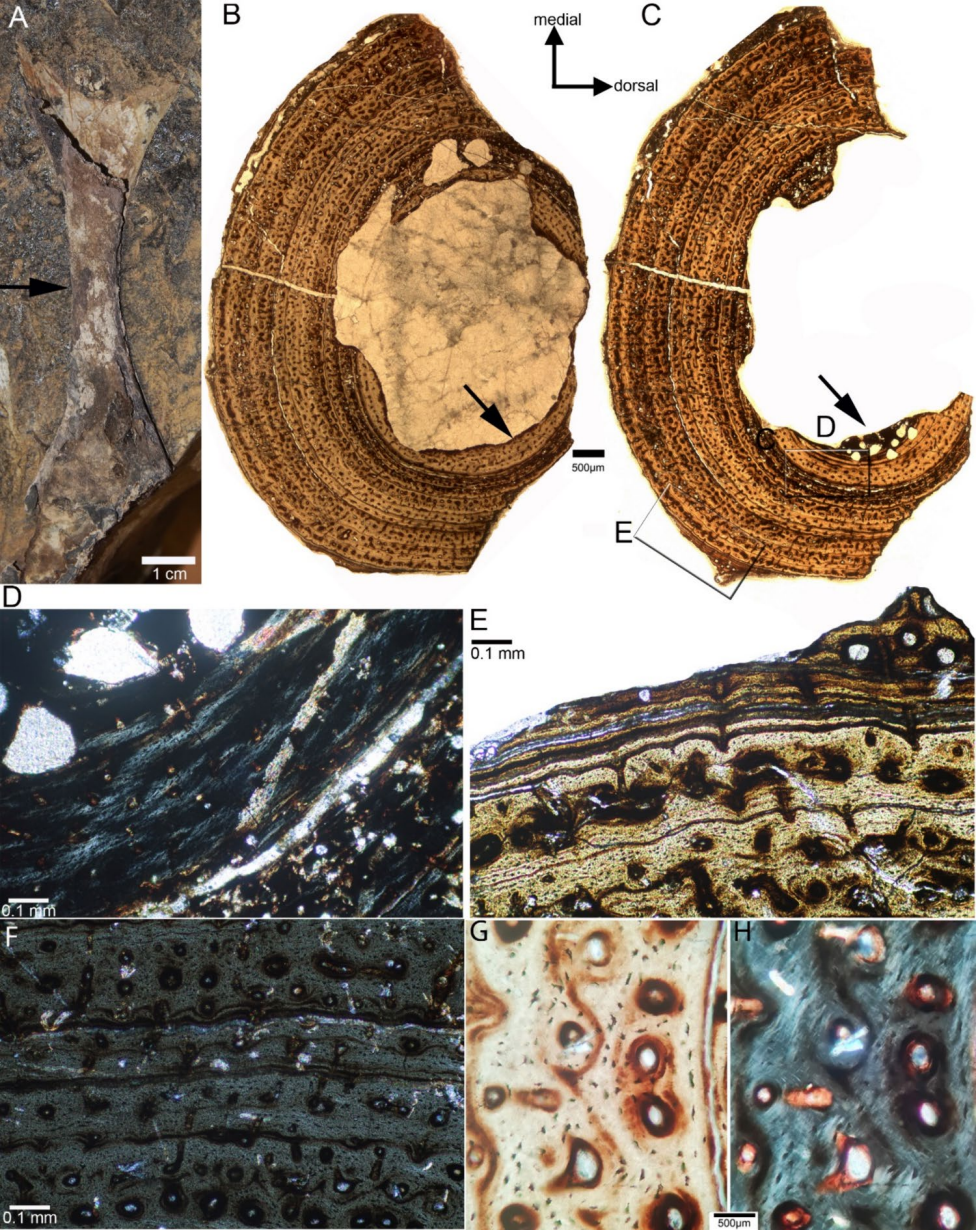
Humeral osteohistology of *Benggwigwishingasuchus eremacarminis*. **A** Original humerus with sampling location marked (arrow). **B** Midshaft section. **C** Section distally to midshaft. Note the remains of an inner ring of higher organized tissue (black arrow in B) and of a medullary region (black arrow in C). **D** Detail of medullary region and subsequently following inner cortex in polarized light. **E** Detail of outer cortex showing closely spaced multiple rest lines with the beginning of another zone on top. **F**–**H** Detail of parallel-fibered matrix, osteocyte lacunae, and primary osteons in polarized and normal light

From the holotype and only known specimen (LACM-DI 158616) of *Benggwigwishingasuchus*, a humeral midshaft section (Fig. 1A) was processed into two petrographic thin sections following the method described in Klein and Sander (2007). The histological study was performed with a Leica DM LP microscope, equipped by an EOS Canon camera. The histological nomenclature follows Buffrénil et al. (2021b). The ratio of the medullary cavity to the periosteal cortex (*k*-value of Currey and Alexander 1985) is 0.44.

## Results

The humeral cross section is incomplete but was originally round oval (Fig. 1B, C). The center of both sections contains interlocking (spiritic) crystals, which are the result of diagenesis. Because crystals usually grew into open spaces, the former presence of a large medullary cavity is likely. An active resorption zone of the inner cortex is indicated by the presence of large erosion cavities scattered around the inner periosteal cortex (Fig. 1B–D). At one corner, round erosion cavities embedded in a matrix of dense endosteal lamellar tissue are visible, indicating the margins of a medullary region here (Fig. 1D).

The cortex of *Benggwigwishingasuchus* shows a parallel-fibered matrix that is locally highly organized and might partially grade into or is intermixed with lamellar-fibered matrix. No woven bone is identified. The tissue of the innermost cortex is more organized than the middle and outer cortex (Fig. 1B, C). The density of tiny osteocyte lacunae is moderate to low. Their shape varies between roundish to spindle-like. Vascularization is low in the innermost cortex and moderately throughout the rest of the cortex. Numerous but small, mainly longitudinally oriented primary osteons are regularly distributed through the cortex (Fig. 1). Some primary osteons are reticularly or radially organized. Primary osteons are arranged in circular rows, parallel to the growth cycles.

The cortex is regularly interrupted by distinct lines of arrested growth (LAG) (Fig. 1). The innermost cortex shows in its inner part a stratification by diffuse thin layers resembling annuli but these are too numerous to represent annual cessations in growth (Fig. 1D). This inner ring is interpreted as the tissue of the earliest preserved ontogenetic stage. It ends in a distinct LAG. The following growth cycles are narrower, vary slightly in thickness but are tendentially regularly spaced. Some cycles end in LAGs accompanied by multiple rest lines.

The outermost cortex shows an accumulation of closely spaced rest lines, followed by a new zone, which is indicated by primary osteons in parallel-fibered matrix (Fig. 1E). Altogether, eight annual growth cycles are counted and a 9th is in the beginning of deposition (Fig. 1E). Counting growth marks does not necessarily reveal a correct age (Schucht et al. 2021), but the high number of LAGs indicates an advanced ontogenetic stage. Without the remains of the initiation of a new growth zone, one could have argued for the presence of an EFS or OCL and a cessation of growth, which is however not the case. The growth rate was slow throughout ontogeny, indicating also a low resting metabolic rate.

## Discussion

The humeral *k*-value (0.44) of *Benggwigwishingasuchus* is higher than those observed in aquatic and semiaquatic animals (*k* = 0.30) but similar to terrestrial forms (Ezcurra et al. 2014). A bone compactness analysis of the femoral midshaft based on μCT data revealed no signal for any aquatic adaptation (Smith et al. 2024). Femoral and humeral microanatomy thus suggest a terrestrial mode of life for *Benggwigwishingasuchus*.

The fiber arrangement in the parallel-fibered tissue in aquatic reptiles is often very coarse indicating an increased growth rate (e.g., Klein 2010) but fiber arrangement is fine in *Benggwigwishingasuchus* (Fig. 1G), which together with the high number of growth marks and the moderate vascularization indicates slow growth rates. The medullary region shows no remains of calcified cartilage. Thus, no secondary aquatic adaptation is identified in osteohistology, supporting a dominantly terrestrial lifestyle for *Benggwigwishingasuchus*. Whether *Benggwigwishingasuchus* was washed into the sea by chance or if it drifted from the coast into the open sea during foraging behavior cannot be clarified. In the latter case, the beginning of adopting a semiaquatic lifestyle was not yet recorded in its skeletal morphology nor in its bone microanatomy or histology.

The histology of several Triassic archosauriform taxa has been studied so far (see Suppl. Table), but no sample matches exactly the histology of another. This is because different skeletal elements and/or different ontogenetic stages have often been studied but mainly due to the high variability in bone tissue types in Triassic archosaurs. However, this high variability might also be related to different environments from where the taxa had been studied as evidenced, e.g., by the osteohistological differences among aetosaurs and phytosaurs from North America (Ricqlès et al. 2003, 2008), South America (Ponce et al. 2022), and Poland (Teschner et al. 2022).

The osteohistology of *Benggwigwishingasuchus eremacarminis* with its scaffold of primary osteons in moderately vascularized parallel fibered and lamellar-fibered matrix resembles that of typical crocodylomorph taxa (Botha et al. 2023). However, osteohistology bears only a poor phylogenetic signal (Ricqlès et al. 2008). *Benggwigwishingasuchus* was recovered as representing the sister-taxon to an extended Poposauroidea (Smith et al. 2024). *Sillosuchus*, a large bipedal poposaurid, shows uninterrupted woven-parallel complex throughout its ontogeny (Curry Rogers et al. 2024). In early ontogeny, the medium-sized *Effigia* (Nesbitt 2007), a bipedal, toothless poposaurid, shows woven-parallel complex followed by well vascularized parallel fibered matrix that finally switches to avascular lamellar tissue (Nesbitt 2006), resulting in a medium fast growth rate. Thus, the growth rates of *Effigia* and *Sillosuchus* were much faster than in *Benggwigwishingasuchus.*

Although there is no endothermic or ectothermic bone tissue per se (Buffrénil et al. 2021a), the bone tissue type can allow inferences about metabolism (Montes et al. 2007; Legendre et al. 2016). Bone cortices mainly composed of parallel-fibered matrix are usually considered to have belonged to a poikilothermic ectotherm (Cubo et al. 2017). The ectothermic condition can reliably be inferred for *Benggwigwishingasuchus.* The numerous resting lines throughout the cortex indicate a growth strategy highly dependent on environmental conditions and in this case can give insights into a strong seasonality of this environment. In addition, a minimum of eight years for such a small individual that is still growing also indicates a low growth rate and thus is consistent with a low basal metabolic rate.

The reason why bone tissue types and inferred growth rates and thus life history strategies in Triassic archosaurs are so variable remains for now unknown but point to a high plasticity. Growing slowly or fast must have provided an advantage under certain environmental conditions within a given body size range.

Generalized inferences about growth and metabolism within Triassic archosaurs are not possible with the current data. However, *Benggwigwishingasuchus* is an important datapoint for future analyses of life history strategies and their constraints in Triassic archosauriformes.

### Supplementary Information

Below is the link to the electronic supplementary material.

Supplementary file1 (DOCX 40 KB)
