# Supplementary material for: Diverse growth rates in Triassic archosaurs—insights from a small terrestrial Middle Triassic pseudosuchian
Source: Naturwissenschaften. 2024 Jul 11;111(4):38. doi: 10.1007/s00114-024-01918-4 (PMC11239758; doi:10.1007/s00114-024-01918-4)
Supplement: Supplementary file 1 — Supplementary file1 (DOCX 40 KB) [file 114_2024_1918_MOESM1_ESM.docx]

Supplementary Table

**Literature overview of tissues in Triassic archosaurs.**

The focus of this table is on bone tissue, indicating fast or slow growth rates. The table is ordered after phylogeny and stratigraphy. For phylogenetic trees plotting tissue types see Klein et al. (2017) and Botha et al. (2023). If not mentioned otherwise the taxa are dominantly terrestrial. Choristodera and Protorosauria are not considered in the current compilation.

The histology of aetosaurs and phytosaurs from localities of North America (Ricqlès et al 2003, 2008) and south America (Ponce et al. 2022) in comparison with close relatives from Poland (Teschner et al. 2022) shows that the environment might have a strong influence on deposited tissue and thus growth rate.

Abbreviations: EFS, external fundamental system; lfb, lamellar-fibred bone; pfb, parallel-fibred tissue; complex; wpc, woven-parallel complex.

| **Taxon** | **Tissue** | **Body size** | **Stratigraphy** | **Group** | **Reference** |
| --- | --- | --- | --- | --- | --- |
| *Prolacerta* | uninterrupted poorly vascularizedwpc & pfb | small-sized | Early Triassic;  S-Africa  (Karoo Fm) | archosauromorph | Botha-Brink and Smith 2011 |
| *Azendhosaurus*  herbivorous | wpc & pfb in hu & fe:  but see various tissues in other elements | medium-  sized  (2-3m) | Late Triassic;  Morocco, Madagascar | archosauromorph | Cubo and Jalil 2019 |
| *Trilophosaurus*  herbivorous/  durophageous | pfb, lfb | medium- sized  (2-3m),  quadruped | Late Triassic;  North America | archosauromorph | Werning and Irmis 2011 |
| *Euparkeria* | cyclical pfb | small-sized | Early to Middle Triassic;  S-Africa  (Karoo Fm) | archosauriform | Botha-Brink and Smith 2011 |
| *Erythrosuchus* | uninterrupted wpc | small- to medium-  sized | Early to Middle Triassic;  Karoo ecosystem | archosauriform | Botha-Brink and Smith 2011 |
| *Chanaresuchus*  semiaquatic | wpc  >. pfb/lfb | small-sized  (1-1.5m) | Middle Triassic (Anisian); Argentina (Chañares Fm) | archosauriform,  proterochampsid | Ricqlès et al. 2008 |
| *Tropidosuchus* | wpc  > pfb/lfb | ?medium-  sized | Middle Triassic (Anisian) Argentina (Chañares Fm) | archosauriform,  proterochampsid | Marsà et al. 2020 |
| *Proterosuchus/*  *Chasmatosaurus* | uninterrupted radial wpc; outer cortex: pfb/lfb | medium-sized  (3.5m),  quadruped | Early Triassic;  Karoo ecosystem | archosauriform | Botha-Brink and Smith 2011 |
| *Proterochampsa*  ?aquatic | uninterrupted wpc >  EFS; | medium-sized,  quadruped | Late Triassic;  (Ischigualasto Fm) | archosauriform | Currey-Rogers et al. 2023 |
| *Pseudochampsa*  ?aquatic | cyclical pfb & lfb, | medium-sized,  quadruped | Late Triassic;  (Ischigualasto Fm) | archosauriform | Currey-Rogers et al. 2023 |
| *Vancleavea*  semiaquatic | cyclical pfb/lfb | small-sized  (1.2m) | Late Triassic;  North America | archosauriform | Nesbitt et al. 2009 |
| *Stenaulorhynchus*  herbivorous | cyclical pfb/lfb | small- to large-sized  (1-6m),  quadruped | Middle Triassic;  Tanzania  (Manda Fm) | archosauromorph,  rhynchosaur | Werning and Nesbitt 2016 |
| *Hyperodapedon*  herbivorous | cyclical wpc alternating with pfb | small-sized | Late Triassic;  Argentina  (Ischigual. Fm),  Brazil  (Santa Maria Fm) | archosauromorph,  rhynchosaur | Veiga et al. 2015;  Currey-Rogers et al. 2023 |
| *Teyumbaita*  herbivorous | cyclical wpc in early onto. wpc  > late onto. cyclical pfb/lfb | ?small-sized | Late Triassic;  Brazil | archosauromorph,  rhynchosaur | Veiga et al. 2015 |
| *Sillosuchus* | uninterrupted laminar wpc | medium- to large-sized  (3- 10) | Late Triassic;  South America  (Ischigualasto Fm) | archosaur,  poposaurid | Currey-Rogers et al. 2023 |
| *Effigia*  toothless | cyclical wpc> late onto. pfb/lb/EFS | medium-sized  (2m) | Late Triassic;  New Mexico  Ghost Ranch | archosaur,  poposaurid | Nesbitt 2007 |
| *Benggwigwishingasuchus* | cyclical pfb/lfb, | small-sized  (1-1.5m) | Middle Triassic;  Nevada  (Prida Fm) | archoaur,  poposauroid | this paper |
| phytosaurs/  aetosaurs | early onto. cyclical wpc  > late onto. pfb/lfb | medium- to large-sized | Late Triassic;  North America | archosauriform/ archosaur | Ricqlès et al. 2003, 2008 |
| aetosaurs and phytosaurs | cyclical pfb/lb  little wb but no wpc | medium- to large-sized | Late Triassic; Poland | archosauriform/ archosaur | Teschner et al. 2022 |
| *Aetosauroides scagliai* | early onto.: cyclical  wpc  > late onto.: pfb/lfb | medium-sized  (2.4 m),  quadruped | Late Triassic;  South America  (Ischigualasto Fm, Santa Maria Fm) | archosaur,  ,  aetosaur | Ponce et al. 2022 |
| *Prestosuchus* | cyclical laminar pfb | large-sized  (5-7m), quadruped | Middle Triassic;  Brazil  (Santa Maria Fm) | pseudosuchian, | Ponce et al. 2023 |
| *Decuriasuchus quartacolonia* | early onto. uninterrupted wpc >  late onto. unknown | medium-sized  (2.5 m) | Middle Triassic/  Ladinian,  Brazil  (Santa Maria Fm) | pseudosuchian | Dantas Macedo de Farias et al. 2023 |
| *Batrachotomus* | cyclical laminar  wpc | large-sized  (3-4 m) | Middle Triassic;  Germany | pseudosuchian | Klein et al. 2017 |
| *Luperosuchus* | early onto. wpc  > late onto. pfb/lfb | large-sized  (4m) | Late Triassic;  Argentina (Chañares Fm) | pseudosuchian | Ricqlès et al. 2008 |
| *Fasolasuchus* | cyclical laminar wpc | large-sized  (8m),  quadruped | Late Triassic;  Argentina  (Los Colorados Fm) | pseudosuchian | Ponce et al. 2023 |
| *Saurosuchus* | cyclical laminar wpc  > late onto. pfb | large-sized  (7m) | Late Triassic;  South America  (Ischigualasto Fm) | pseudosuchian | Currey-Rogers et al. 2023 |
| *Postosuchus* | wpc > late onto pfb/lfb/ EFS | medium-sized | Late Triassic;  USA  Dockum Group | pseudosuchian | Ricqlès et al. 2003 |
| *Trialestes* | uninterrupted wpc > late onto. pfb/lfb | **?** | Late Triassic;  South America  (Ischigualasto Fm) | crocodylomorphs | Currey-Rogers et al. 2023 |
| *Gracilisuchus* | cyclical pfb/lfb | small-sized (30cm),  biped | Late Triassic;  Argentina | close to the ancestry of crocodylomorphs | Lecuona et al. 2020 |
| *Hesperosuchus* | cyclical pfb/lfb | small-sized  (1.5m) | Late Triassic;  Arizona,  New Mexico | crocodylomorph | Ricqlès et al. 2003; |
| *Terrestrisuchus* | pfb/lfb | small-sized | Late Triassic;  England | crocodylomorph | Ricqlès et al. 2003;  Cubo et al. 2017 |
| *Sphenosuchus* | cyclical growth, early onto. wpc  > late onto pfb/lfb | small-sized  (1.5m) | Early Jurassic;  S-Africa  (Elliot Fm) | archosaur,  crocodylomorph | Botha et al. 2023 |
| *Iberosuchus* | cyclical growth, pfb/lfb,  but with one layer of radial wpc | medium-  sized | Middle Eocene;  Europe | sebecosuchian meso-eucrocodylian | Cubo et al. 2017 |

**References for Suppl. Tab**

Botha J, Weiss BM, Dollman K, Barrett PM, Benson RBJ, Choiniere JN (2023) Origins of slow growth on the crocodilian stem lineage. Curr Biol 33(19):4261–4268.e3 doi.org/10.1016/j.cub.2023.08.057.

Botha-Brink J, Smith RMH (2011) Osteohistology of the Triassic archosauromorphs *Prolacerta*, *Proterosuchus*, *Euparkeria*, and *Erythrosuchus* from the Karoo Basin of South Africa. J Vert Paleontol 31:1238–1254.

Cubo J, Jalil N-E (2019) Bone histology of *Azendohsaurus laaroussii*: implication for the evolution of thermometabolism in archosaumorpha. Paleobiology 45:317–330.

Cubo J, Köhler M, Buffrenil V de (2017) Bone histology of *Iberosuchus macrodon* (Sebecosuchia, Crocodylomorpha). Lethaia 50:495–503. https://doi.org/10.1111/let.12203

Curry-Rogers K, Martínez RN, Colombi C, Rogers RR, Alcober O (2024) Osteohistological insight into the growth dynamics of early dinosaurs and their contemporaries. PLoS ONE 19(4): e0298242. <https://doi.org/10.1371/journal.pone.0298242>.

Dantas Macedo de Farias B, Desojo JB, Cerda IA, Ribeiro AM, Ferigolo J, Carlisbino T, Schultz CL, Mastrantonio B, and Bento Soares M (2023) Bone histology supports gregarious behavior and an early ontogenetic stage to *Decuriasuchus quartacolonia* (Pseudosuchia: Loricata) from the Middle-Late Triassic of Brazil. The Anatomical Record <https://doi.org/10.1002/ar.25365>.

Ezcurra MD, Scheyer TM, Butler RJ (2014) The origin and early evolution of Sauria: reassessing the Permian saurian fossil record and the timing of the crocodile-lizard divergence. PLoS ONE 9:e89165.

Klein N, Foeth Ch, Schoch RR (2017) Preliminary observations on the bone histology of the Middle Triassic pseudosuchian archosaur *Batrachotomus* *kupferzellensis* reveal fast growth with laminar fibrolamellar bone tissue. J Vert Pal 37(4): https://doi.org/10.1080/02724634.2017.1333121

Lecuona A, Desojo JB, Cerda IA (2020) New information on the anatomy and histology of *Gracilisuchus stipanicicorum* (Archosauria: Pseudosuchia) from the Chañares Formation (early Carnian), Argentina. C R Palevol 19(3):40–62. https://doi.org/10.5852/cr-palevol2020v19a3.

Garcia Marsà, JA, Ponce DA, Agnolin FL, Novas FE (2020) Histovariability and lifestyle in Proterochampsidae Romer, 1966 (Archosauriformes) from the Chañares Formation (Late Triassic), northwestern Argentina. C R Palevol 22(30):605–622.

Nesbitt SJ (2006) The anatomy of *Effigia okeeffeae* (Archosauria, Suchia),

theropod-like convergence, and the distribution of related taxa. Bull Am Mus Nat Hist 302:1–84.

Nesbitt SJ, Stocker MR, Small BJ, Downs A (2009) The osteology and relationships of *Vancleavea campi* (Reptilia: archosauriformes). Zool J Linn Soc 157:814–864.

Ponce DA, Desojo JB, Cerda IA. (2022) Palaeobiological inferences of the aetosaur *Aetosauroides scagliai* (Archosauria: Pseudosuchia) based on microstructural analyses of its appendicular bones. Hist Biol. doi.org/10.1080/08912963.2022.2035728.

Ponce DA, Scheyer TM, Cerda IA, Desojo JB (2023) Palaeobiological inferences of

“rauisuchians” Fasolasuchus tenax (Los Colorados Fm., Argentina) and *Prestosuchus chiniquensis* (Santa Maria Super sequence, Brazil) from the Middle–Upper

Triassic of South America based on microstructural analyses. J Anatom, 00:1–17. <https://doi.org/10.1111/joa.13937>

Ricqlès A de, Padian K, Horner JA (2003) On the bone histology of some Triassic pseudosuchian archosaurs and related taxa. Ann Palaeontol 89:67–101.

Ricqlès A de, Padian K, Knoll F, Horner J. A (2008) On the origin of high growth rates in archosaurs and their ancient relatives: complementary histological studies on Triassic archosauriforms and the problem of a “phylogenetic signal” in bone histology. Ann Palaeontol 94:57–76.

Teschner E, Konietzko-Meier D, Klein N (2022). Growth and limb bone histology of

aetosaurs and phytosaurs from the Late Triassic Krasiejów locality (sw Poland) reveals strong

environmental influence on growth pattern. Contrib Zool 91:199–232.

Veiga FH, Soares MB, Sayão JM (2015) Osteohistology of hyperodapedontine rhynchosaurs from the Upper Triassic of Southern Brazil. Acta Palaeont Pol 60:829–836.

Werning S, R. B. Irmis (2010) Reconstructing the ontogeny of the Triassic basal archosauromorph using bone histology and limb bone morphometrics. J Vert Paleont 30 (Suppl.):185A–186A.

Werning S, Irmis RB (2011) Reconstructing growth of the basal archosauromorph *Trilophosaurus*. Integr Comp Biol 51:SICB 2011 Annual Meeting, Salt Lake City. Abstract 39.2/E147.

Werning S, Nesbitt SJ (2016) Bone histology and growth in *Stenaulorhynchus stockleyi* (Archosauromorpha: Rhynchosauria) from the Middle Triassic of the Ruhuhu Basin of Tanzania. C R Palevol 15(1):163–175. doi:10.1016/j.crpv.2015.03.004
